# Ecological Processes of Bacterial and Fungal Communities Associated with *Typha orientalis* Roots in Wetlands Were Distinct during Plant Development

**DOI:** 10.1128/spectrum.05051-22

**Published:** 2023-01-23

**Authors:** Lixiao Wang, Jinxian Liu, Meiting Zhang, Tiehang Wu, Baofeng Chai

**Affiliations:** a Institute of Loess Plateau, Shanxi University, Shanxi Key Laboratory of Ecological Restoration for Loess Plateau, Taiyuan, China; b Department of Biology, Georgia Southern University, Statesboro, Georgia, USA; University of Sussex

**Keywords:** root-associated microbiome, root endosphere, ecological process, developmental stage, wetland, *Typha orientalis*

## Abstract

Root-associated microbiomes are essential for the ecological function of the root system. However, their assembly mechanisms in wetland are poorly understood. In this study, we explored and compared the ecological processes of bacterial and fungal communities in water, bulk soil, rhizosphere soil, and root endosphere niches for 3 developmental stages of *Typha orientalis* at different wetland sites, and assessed the potential functions of root endosphere microbiomes with function prediction. Our findings suggest that the microbial diversity, composition, and interaction networks along the water-soil-plant continuum are shaped predominantly by compartment niche and developmental stage, rather than by wetland site. Source tracking analysis indicated that *T. orientalis’* root endosphere is derived primarily from the rhizosphere soil (bacteria 39.9%, fungi 27.3%) and water (bacteria 18.9%, fungi 19.1%) niches. In addition, we found that the assembly of bacterial communities is driven primarily by deterministic processes and fungal communities by stochastic processes. The interaction network among microbes varies at different developmental stages of *T. orientalis*, and is accompanied by changes in microbial keystone taxa. The functional prediction data supports the distribution pattern of the bacterial and fungal microbiomes, which have different ecological roles at different plant developmental stages, where more beneficial bacterial taxa are observed in the root endosphere in the early stages, but more saprophytic fungi in the late stages. Our findings provide empirical evidence for the assembly, sources, interactions, and potential functions of wetland plant root microbial communities and have significant implications for the future applications of plant microbiomes in the wetland ecosystem.

**IMPORTANCE** Our findings provide empirical evidence for the assembly, sources, interactions, and potential functions of wetland plant root microbial communities, and have significant implications for the future applications of plant microbiomes in the wetland ecosystem.

## INTRODUCTION

Plants cohabit with diverse microbes in nature. Healthy and asymptomatic plants and plant-associated microbiomes can form a complex “holobiont” that plays an important role in the function and adaptability of plants on both an ecological and evolutionary time scale (1, 2). The plant microbiome acts as a secondary genome, which is linked to plant growth and development, nutrient absorption, disease resistance, and environmental stress adaptation (3, 4). The plant can modulate its microbiota to dynamically adjust to its environment (5, 6). Studies have found that plants harbor distinct microbiota that are a subset of those present in their surrounding environment. However, the assembly mechanism determining the endophytic microbial community remains unclear.

The roots system serves as a hot spot of plant-environment-microbes interaction and, plays direct roles in plant growth, health, and adaptability (7). The microbial community in plant roots is primarily divided into rhizosphere and endosphere microbial communities according to their habitats around and inside the roots, respectively (8). Bulgarelli et al. proposed a two-step model for root microbiome assembly (9). First, rhizosphere deposition drives microbes to migrate from bulk soil to the rhizosphere, and form rhizosphere microflora. Second, rhizosphere microbes enter the root endosphere under the control of the host genotype, and establish rhizosphere microflora. Root microbial communities are primarily derived from the horizontal migration of bulk soil microbes, leading to bulk soils being considered the “seed bank” of root microbial communities (5, 10). The root microbial community can also be considered a subset of the bulk soil microbial community, formed by a filtration process (11). Recent studies on the endosphere and rhizosphere have shown that abiotic stresses in the soil environment and biotic factors in the host plants affect the diversity and abundance of root-associated communities (12–15). Furthermore, plant species, genotype, and developmental stage affect diversity, and shape the composition of root-associated microbiomes (16). In particular, as the root matures, changes in the rhizosphere and endosphere communities lead to functional shifts in the microbial community. While evidence on the dynamics of root-associated microbial communities has been accumulating, a comprehensive understanding of the temporal dynamics of microbial community functions in the root endosphere is still missing.

Biotic and abiotic factors are known to influence plant microbiome assembly (17, 18), and it is generally accepted that deterministic and stochastic processes jointly determine the assembly of microbial communities. Several studies have used the null model theory to understand the assembly mechanisms of microbial communities in plant roots (19–21). For example, bacterial and fungal communities were dominated by deterministic factors in both bulk soil and wheat rhizosphere. In addition, Fan et al. found that the importance of deterministic processes in determining diazotrophic communities decreased with distance from wheat roots (19). However, the relative importance of ecological processes, such as heterogeneous selection, homogeneous selection, homogeneous dispersal, dispersal limitation, and undominated processes that govern the composition of root-associated microbial communities, remain poorly understood. In addition to plant and environmental factors, the assembly of the root-associated microbial communities is strongly influenced by microbe-microbe interactions (22–24). Co-occurrence network analyses have been used to explore potential direct or indirect interactions among microbial species. Highly connected microbial taxa within the network possibly exert a considerable impact on the microbiome, regardless of their abundance, and are proposed to be keystone taxa (25). For example, by examining the effect of the keystone microbe Enterobacter cloacae on corn root microbiota, it was determined that the presence of a keystone microbe could have a large effect on community assembly (8). However, the ecological function of root-associated microbial keystone taxa remains largely unknown.

Wetlands are different from terrestrial and aquatic ecosystems, where plants are affected by both water and soil environments. Wetland plants and plant-associated microorganisms are major components of the wetland ecosystem, serving important functions in regulating climate and degrading pollutants (26, 27). Most previous studies on plant root-associated microbiomes focused on the terrestrial ecosystem, creating a gap in our knowledge concerning microbial colonization of wetland plant roots. Notably, our understanding of bacterial-fungal interactions along the water-soil-plant continuum and how they respond to changes across plant developmental stages remains unclear.

In this study, we investigated the bacterial and fungal communities of *Typha orientalis* over 3 developmental stages (seeding, mature, and wilting) across 162 samples from water, bulk soil, rhizosphere soil, and root endosphere niches. We sought to: (i) illuminate the mechanisms and ecological processes of *T. orientalis* root-associated microbiome assembly, and the interaction of these microbiomes by compartmental niche, plant development, and environmental factors; (ii) clarify the keystones of *T. orientalis* root-associated microbiome and their ecological functions at different stages. We hypothesized that: (i) waterborne microbes are one source of the root endosphere; (ii) The assembly mechanisms and ecological processes of bacterial and fungal communities are different; (iii) The keystone taxa of root-related microbial communities will change, and play distinct ecological roles across plant developmental stages.

## RESULTS

### Diversity and structure of microbial communities.

**FIG 1:**
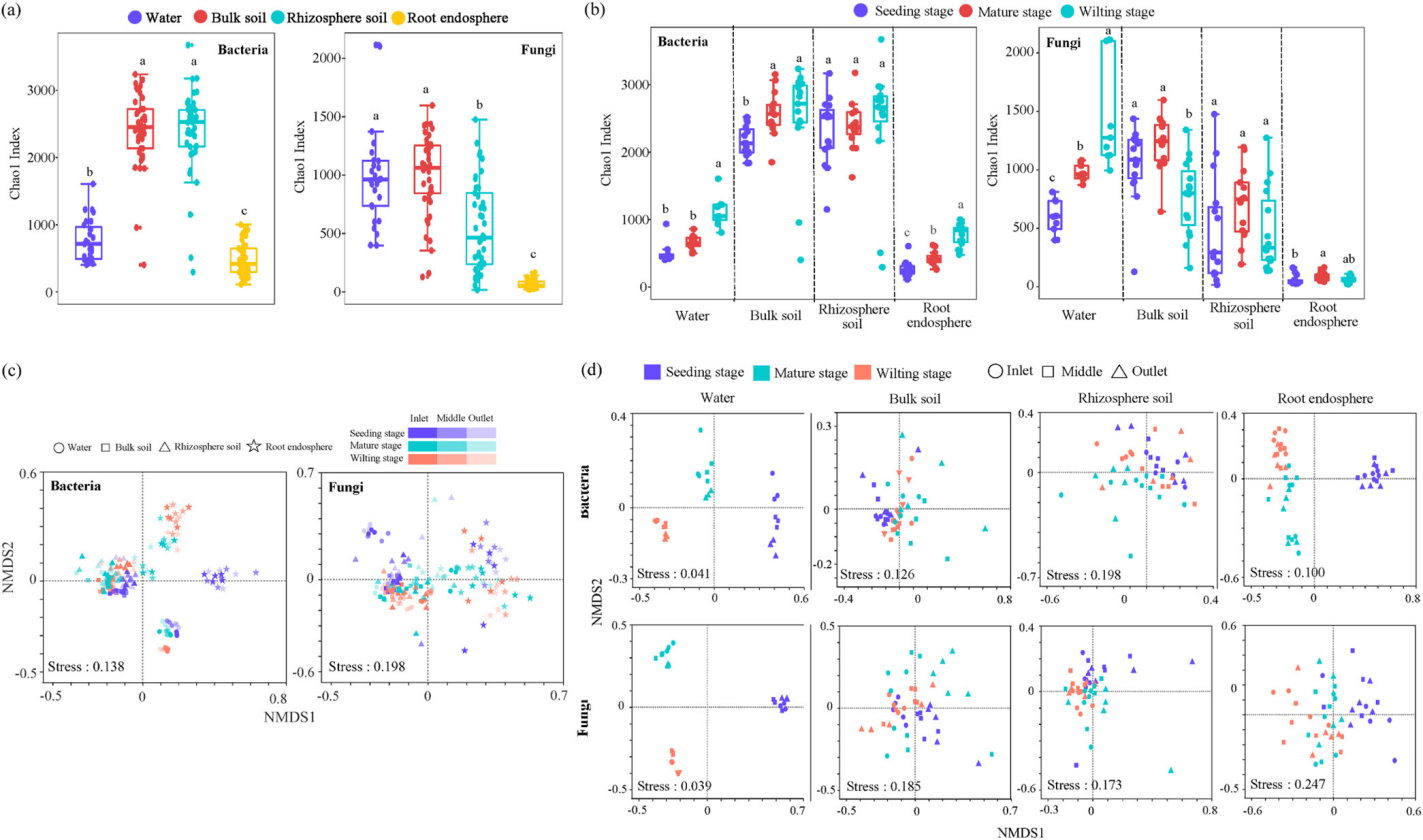
Temporal dynamics of diversity and distribution patterns of microbiomes. (a) Alpha diversity of bacterial and fungal communities in water, bulk soils, rhizosphere soils, and root endosphere. (b) Alpha diversity of bacterial and fungal communities in water, bulk soils, rhizosphere soils, and root endosphere across 3 plant developmental stages. (c) NMDS ordinations based on Bray-Curtis dissimilarity matrices depicting the distribution patterns of bacterial and fungal communities along the water-soil-plant continuum (*n* = 162). (d) NMDS ordinations based on Bray-Curtis dissimilarity matrices of bacterial and fungal communities in each compartment niche (water: *n* = 27; other niches: *n* = 45).

A total of 11,728 bacterial operational taxonomic units (OTUs) and 11,188 fungal OTUs with > 97% similarity were clustered from 13,448,429 high-quality 16S rRNA reads and 13,960,302 high-quality ITS reads, respectively, across all samples. Multifactorial ANOVA analyses showed that compartment niche, plant developmental stage, and sampling site had different effects on bacterial and fungal alpha diversity, and changes in compartment niche and developmental stage were significant contributors to alpha diversity (*P *<* *0.01) (Table S4). Furthermore, there were significant differences in the diversity (Chao1 index) of bacterial and fungal communities among the compartment niches of water, bulk soil, rhizosphere soil, and root endosphere (*P *<* *0.05). We observed the lowest Chao1 richness in the root endosphere, and the highest in bulk soil for both bacterial and fungal communities (*P *<* *0.05) (Fig. 1a). For compartment niches, plant developmental stage had a greater influence on both bacterial and fungal Chao1 richness in water and root endosphere than in the rhizosphere soil (*P *<* *0.05) (Fig. 1b and Table S4). In bulk soil, developmental stage had a significant effect on fungal Chao1 richness, but no significant effect on bacterial Chao1 richness (*P *<* *0.01) (Table S4). The sampling site only had a significant impact on fungal Chao1richness in niches of water and root endosphere (*P *<* *0.01) (Table S4). Altogether, microbial diversity along the water-soil-plant continuum was related to their niche and plant developmental stage, but the sampling site had little influence.

Nonmetric multidimensional scaling (NMDS) ordinations and PERMANOVA analyses indicated that compartment niche explained the largest variations (17.2 to 37.9%) in both bacterial and fungal communities among all samples, followed by plant developmental stage (6.6 to 7.7%) (Fig. 1c and Table S5). For each compartment niche, plant developmental stage had significant influence on both bacterial and fungal communities, with the strongest effect in the water (bacteria 77.5%, fungi 69.3%) (Fig. 1d and Table S5). The sampling site explained the variations (7.2 to 15.1%) of bacterial and fungal communities in bulk and rhizosphere soil. In contrast, sampling site had no significant effect on bacterial and fungal communities in the water and root endosphere samples (Fig. 1c and d and Table S5). Generally, our findings indicated that the diversity and structure of bacterial and fungal communities were affected by compartment niches, plant developmental stages, and sampling sites.

### Composition and source of microbial communities.

**FIG 2:**
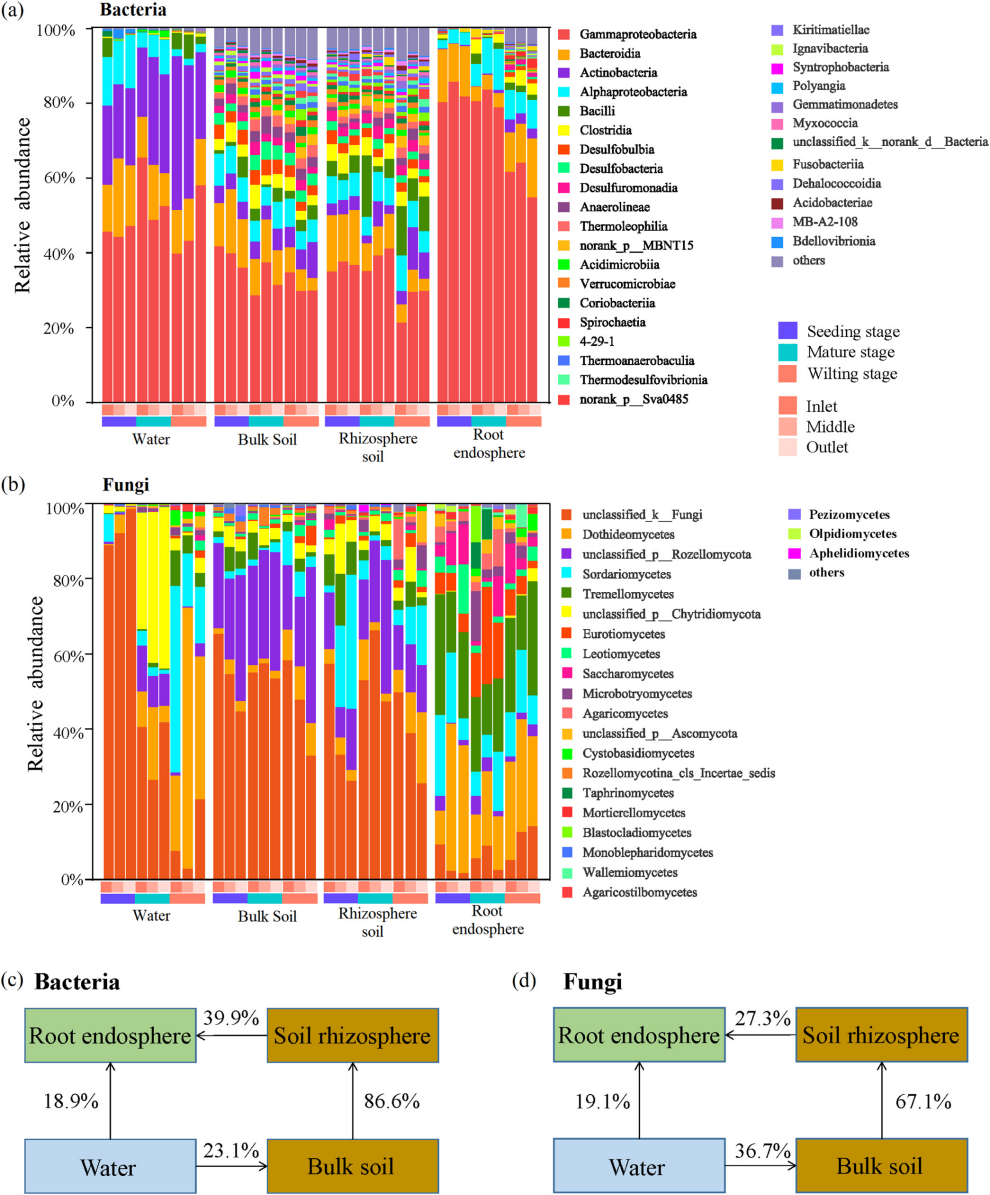
Taxonomic composition and potential sources of bacterial and fungal microbiomes. Temporal dynamics of (a) bacterial communities and (b) fungal communities in water, bulk soils, rhizosphere soils, and root endosphere at 3 sampling sites. Potential source and contribution of the root-associated (c) bacterial community and (d) fungal community.

The relative abundance of bacterial and fungal classes differed significantly across the 4 compartment niches (water, bulk soil, rhizosphere soil, and root endosphere). In general, Gammaproteobacteria, Bacteroidia, and Alphaproteobacteria were the most abundant bacterial classes across all compartments, and Dothideomycetes, Rozellomycota, and Sordariomycetes were the most abundant fungal classes (Fig. 2). For each compartment niche, we found that both bacterial and fungal dominant classes varied distinctly across the three developmental stages. For example, the relative abundance of Gammaproteobacteria was higher at the seedling stage in the bulk soil and root endosphere microbiomes compared with all other stages, but in water and rhizosphere soil, it was higher at the mature stage (Fig. S1). Moreover, Actinobacteria were more abundant in the water microbiome and root endosphere at the wilting stage but showed no significant differences among the three developmental stages in bulk soil and rhizosphere soil (Fig. S1). In addition, the relative abundance of Dothideomycetes increased from the seedling stage to the wilting stage in water and bulk soil microbiome, but no changes were observed in rhizosphere soil and root endosphere (Fig. S1). Unclassified_p_Rozellomycota abundances in water and rhizosphere soil were highest at the mature stage. Similarly, Agaricomycetes abundances in rhizosphere soil and root endosphere were highest at the mature stage (Fig. S1). Differential abundance analysis at the OTU level further demonstrated that some OTUs are significantly enriched at each developmental stage in the 4 compartment niches (Fig. S2). *Pseudomonadaceae*, *Flavobacteriaceae*, *Alcaligenaceae*, and other bacterial families’ OTUs were enriched at the seeding stage, particularly in the plant root endosphere. In contrast, *Didymellaceae*, *Sclerotiniaceae*, *Trimorphomycettaceae*, and other fungal families’ OTUs were enriched at the seeding stage (Fig. S2).

To understand the dynamic characteristics of microorganisms in the environment and the plant, we performed source analysis using the Source Tracker method. Our results showed that *T. orientalis’* root endosphere niche was derived primarily from rhizosphere soil (bacteria 39.9%, fungi 27.3%) and water (bacteria 18.9%, fungi 19.1%) niches (Fig. 1c to d). Moreover, most of the microbes in the rhizosphere soil were derived from the bulk soil (bacteria 86.6%, fungi 67.1%) (Fig. 1c and d). Furthermore, waterborne microorganisms are one source of the bulk soil microbiome (bacteria 23.1%, fungi 36.7%) (Fig. 1c and d). Source analysis of each developmental stage showed that the bacterial communities in water and rhizosphere soil contribute more to the bacterial community in the root endosphere at the seedling stage than in the other 2 developmental stages. Similarly, the fungal communities in water and rhizosphere soil contributed more to the fungal community in the root endosphere at the wilting stage than in the other 2 developmental stages (Fig. S3). Interestingly, the bacterial communities in water and rhizosphere soil contributed more to the root endosphere than did their fungal communities.

### Assembly mechanisms of root-associated microbial communities in spatiotemporal continuum.

**FIG 3:**
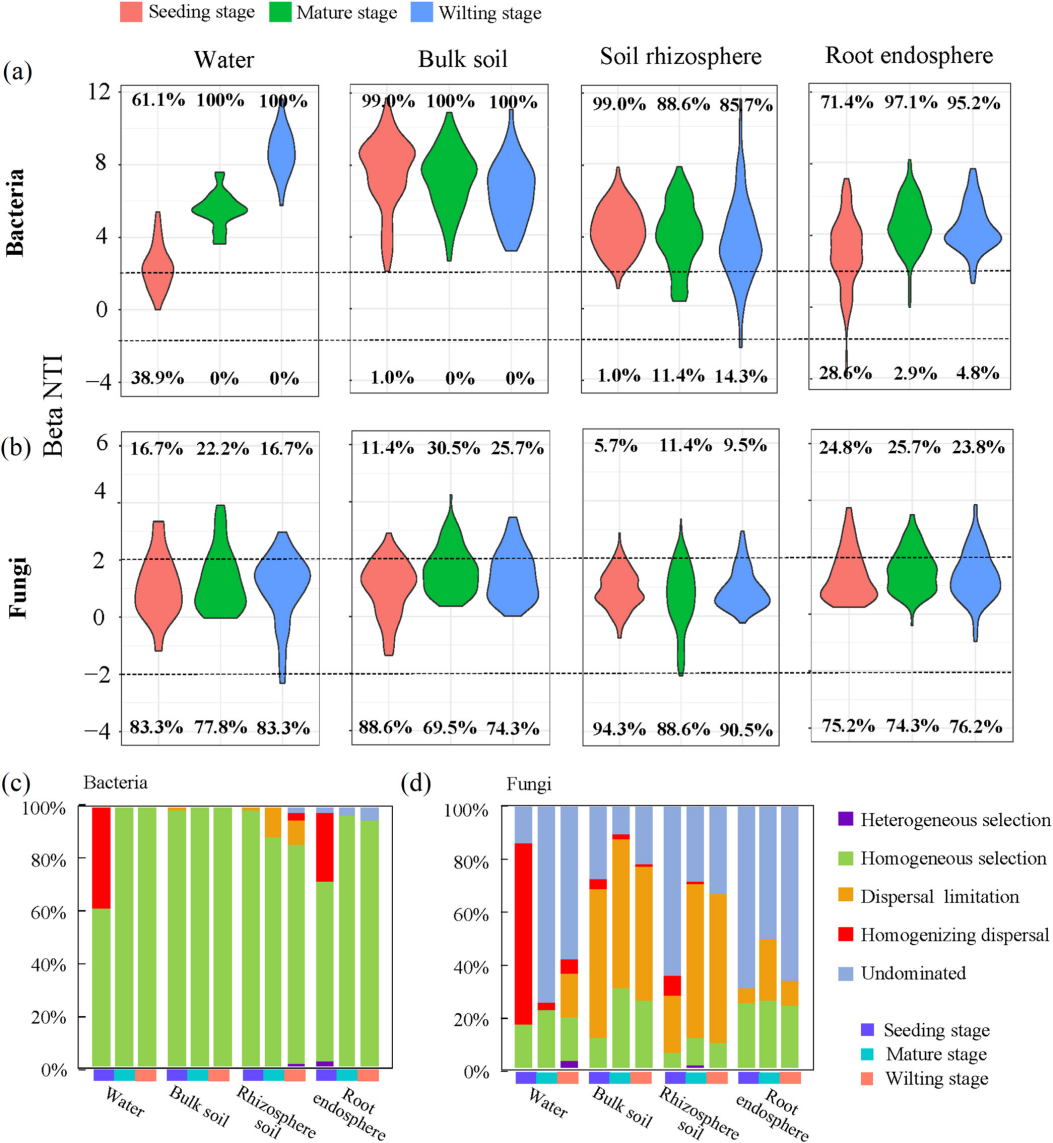
Deterministic and stochastic processes in microbiome assembly. (a and b) Relative contributions of determinism (|βNTI| ≥ 2) and stochasticity (|βNTI| < 2) on microbiome assembly along the water-soil-plant root continuum under a null model. The percentage above and below the violin plot represents the proportion of deterministic and stochastic processes in microbiome assembly, respectively. (c and d) The relative importance of 5 ecological processes along the water-soil-plant root continuum: heterogeneous selection (βNTI < -2), homogeneous selection (βNTI > 2), dispersal limitation (|βNTI| < 2 and RC_Bray_ > 0.95), homogenizing dispersal (|βNTI| < 2 and RCBray < -0.95), and undominated (|βNTI| < 2 and |RC_Bray_| < 0.95).

Using a null model analysis, we found that bacterial community assembly was dominated by the deterministic (beta Nearest Taxon Index |[βNTI]| ≥ 2) process, but fungal community assembly was instead dominated by the stochastic (|βNTI| < 2) process. Plant developmental stage had a greater impact on the relative importance of deterministic and stochastic processes in microbiome assembly in each niche, except for bacteria in bulk soil and fungi in the root endosphere. Different compartment niches also affected the community assembly process (Fig. 3a and b), particularly the higher relative contributions of deterministic processes primarily belonging to heterogeneous selection in rhizosphere soil and root endosphere bacterial communities. The effects of heterogeneous selection processes decreased for bacterial communities in rhizosphere soil with the passage of developmental stages, and the effect of the homogeneous selection process on the root endosphere bacterial community at the seedling stage was weaker than in the 2 other developmental stages. We observed higher relative contributions of stochastic processes belonging to dispersal limitation in bacterial communities of rhizosphere soil at the mature and wilting stages. The homogenizing dispersal process of the bacterial community in rhizosphere soil only existed at the wilting stage and in the root endosphere, only at the seedling stage (Fig. 3c). For the assembly of rhizosphere soil and root endosphere fungal communities, higher relative contributions of stochastic processes belonging to dispersal limitation were found at the mature stage, but the processes were undominated at the seeding stage (Fig. 3d).

As environmental factors can affect plant growth and microbial activity, we performed a Mantel analysis on the relationship between bacterial and fungal communities with environmental factors. We found environmental factors had a greater impact on the water microbiome than on soils and plants. The results showed that bacterial and fungal communities were strongly correlated with temperature (temp), pH, dissolved oxygen (DO), electric conductivity (EC), nitrate nitrogen (NO_3_-N) content, and ammonium nitrogen (NH_4_^+^-N) content (Table S7). In bulk soil, the bacterial community was closely related to NH_4_^+^-N and total phosphorus (TP) content, while the fungal community was related to soil organic carbon (SOC) content. In rhizosphere soil, NH_4_^+^-N content was only significantly correlated with the fungal community. In the root endosphere, sulfur and NH_4_^+^-N content significantly influenced the bacterial community (Table S6). The activities of sucrase, urease, and alkaline phosphatase in rhizosphere soil significantly affected the bacterial communities of rhizosphere soil and root endosphere. However, sucrase and alkaline phosphatase activities in rhizosphere soil only significantly affected the fungal community in rhizosphere soil but not the fungi in the root endosphere (Table S8).

### Co-occurrence networks analysis of microbial communities.

**FIG 4:**
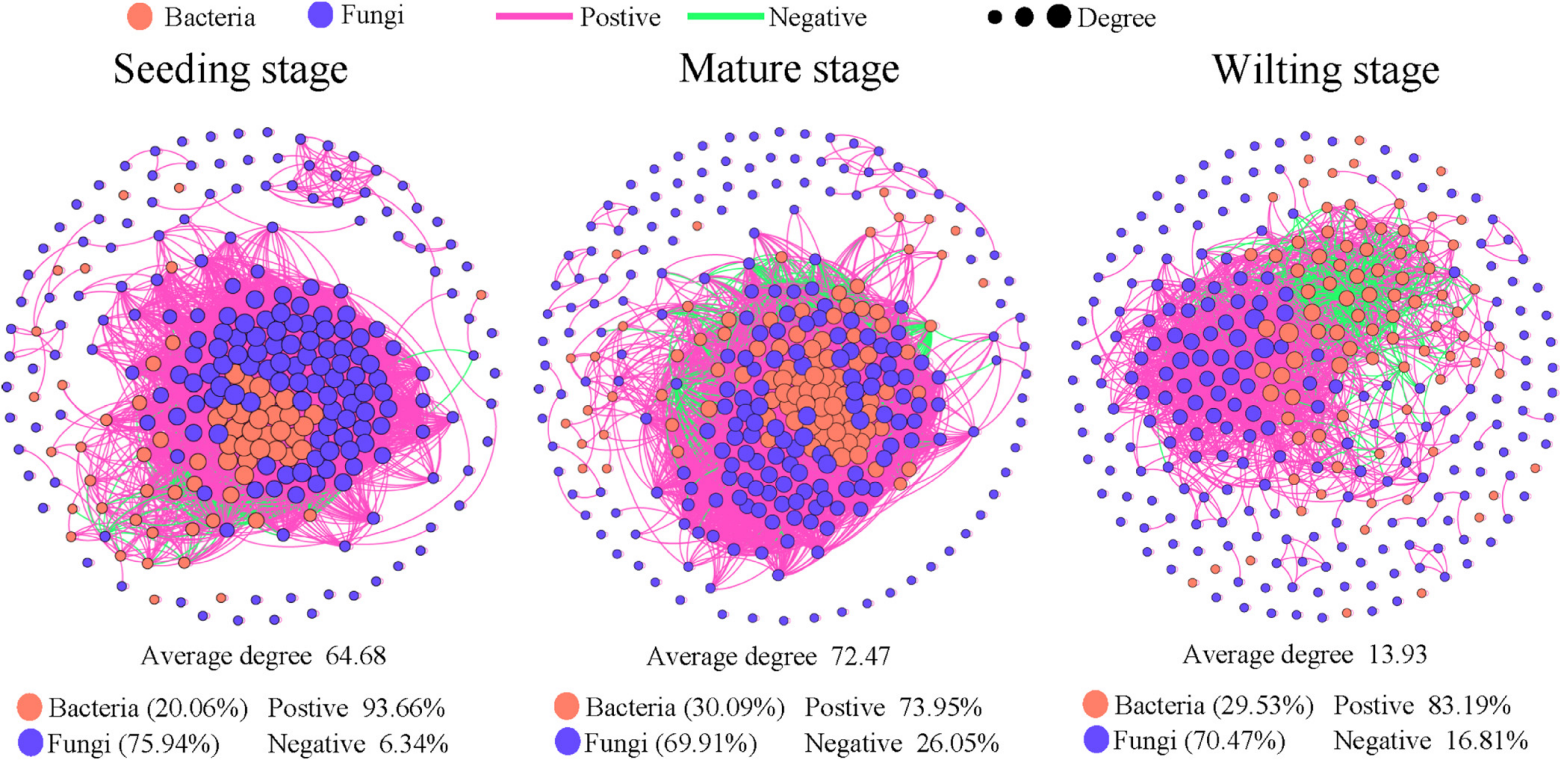
Temporal dynamics of bacterial-fungal interaction networks. Co-occurrence network analysis of the full data set (*n* = 162) of microbial interaction network patterns across the three developmental stages.

The co-occurrence patterns of bacteria and fungi at different developmental stages were explored based on strong significant correlations (*r *>* *0.6 and *P *<* *0.01). Our results showed that the interaction network of the microbiome changed clearly across the 3 developmental stages. The numbers of nodes and edges were greatest at the mature stage, as was the average degree. The number of edges decreased from 8,602 at the seeding stage to 2,076 at the wilting stage. These findings show that the relationship between the microbiome was weakest at the wilting stage. Network correlation was mainly positive. However, with progressing developmental stage, the degree of negative correlation in the wilting stage increased, and then slightly decreased (Table S9). At each developmental stage, the number of network nodes for fungi was more than that for bacteria. However, the degree of network for bacteria was higher than for fungi. The average degree for bacteria was highest at the mature stage, but for fungi, it was instead highest at the seedling stage (Fig. 4 and Table S9).

Finally, we selected the nodes with the highest degree at each developmental stage as the keystones. We found that these keystones of bacterial OTUs for the first 2 developmental stages were more than those of fungal OTU, and the opposite was true for the wilting stage (Table S11). These findings show that, while there were fewer bacterial nodes in the network, there were more bacterial keystones in the seedling and mature stages. The main bacterial nodes with a high network degree were Alphaproteobacteria, Actinobacteria, Verrucomicrobiae, Desulfobulbia, and Gammaproteobacteria at the seeding stage, Gammaproteobacteria, Desulfobulbia, and Thermoleophilia at the mature stage, and Gammaproteobacteria at the wilting stage (Table S11). The main fungal nodes with a high network degree were Rozellomycota at the seeding and wilting stages (Table S11). These results show that there are different keystones at different plant developmental stages.

For each niche, the changes in nodes and edges across developmental stages were consistent with the overall network. In particular, the negative correlation in the water network is greater than in others, as was the positive correlation in root endosphere networks. Moreover, modularity is more significant in the root endosphere networks (Fig. S4 and Table S10). However, we found that most keystones in the root endosphere were fungal OTUs, regardless of developmental stage (Table S12). The main fungal keystones at the class level were: Eurotiomycetes, Saccharomycetes, Sordariomycetes, and Desulfobulbia at the seeding stage; Agaricomycetes, Eurotiomycetes, Tremellomycetes, and Dothideomycetes at the mature stage; and Dothideomycetes at the wilting stage.

### The functional profiles of root endosphere microbial communities.

**FIG 5:**
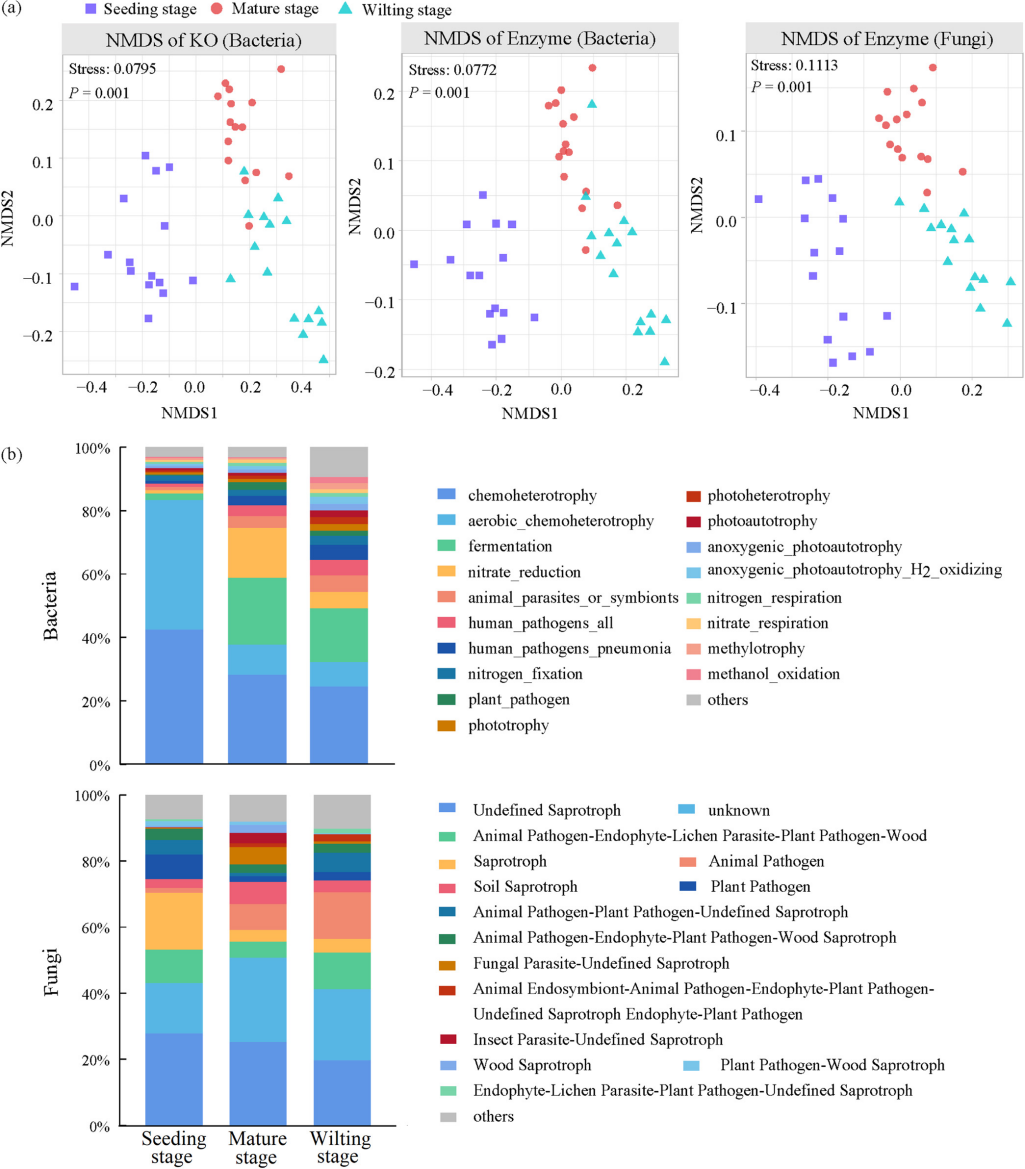
Functional profiles of root endosphere microbiomes. (a) NMDS ordinations based on Bray-Curtis dissimilarity matrices of functional taxa in bacterial and fungal communities in the root endosphere across the 3 plant developmental stages (*n* = 45). (b) The temporal dynamics of functional taxa compositions of bacterial and fungal communities based on FAPROTAX (bacteria) and FUNGuild (fungi) in the root endosphere.

To explore the role of plant roots in the developmental stages, we analyzed the functions of microorganisms in the root endosphere using functional prediction. PICRUSt2 of functional annotation analysis indicated 7,289 KEGG Orthologies (KO), 2,307 enzymes in bacteria, and 901 enzymes in fungi. We used NMDS analyses with the Bray–Curtis distance derived from KO and the enzymes to explore ecological functional clustering based on the different stages. The results revealed a separation of the 3 developmental stages (Stress < 0.2, ANOSIMR; *P *=* *0.001) (Fig. 5a), indicating significant differences in function among the different developmental stages. In addition, we used the FAPROTAX tool to annotate the functions of bacterial communities, obtaining 52 functional groups, of which the most abundant were chemoheterotrophy, aerobic chemoheterotrophy, fermentation, and nitrate reduction (Fig. 5b). The result showed that functional groups were significantly different among the 3 developmental stages. For example, functional groups related to the nitrogen cycle, such as nitrate reduction and respiration, and nitrogen respiration, were more abundant at the mature stage (Fig. S5).

Finally, we used the FUNGuild tool to annotate the functions of fungal communities, obtaining 72 functional groups, of which the most abundant were undefined saprotroph, animal pathogen-endophyte-lichen, parasite-plant, pathogen-wood, saprotroph, animal pathogen, and soil saprotroph (Fig. 5b). The result showed that functional groups, such as animal pathogen, soil saprotroph, and wood saprotroph, had significant differences among the 3 developmental stages. In particular, soil saprotrophs were more abundant at the wilting stage, and wood saprotrophs were more abundant at the mature stage (Fig. S6).

## DISCUSSION

We experimentally studied the spatiotemporal dynamics of the root-associated microbiome of field-grown *T. orientalis* in compartments along the water-soil-plant continuum, and across plant developmental stages, revealing the assembly mechanisms underlying bacterial and fungal communities. Our results provide compelling empirical evidence that both water and soil contribute to the assembly of *T. orientalis* root-associated microbiomes, and that plant developmental stage has a greater impact than sampling site on microbial communities. Crucially, bacterial and fungal communities showed different responses during plant development. Altogether, these results confirmed our hypotheses.

### Determining factors of microbial communities associated with the *T. orientalis* root.

We found that compartment niche, plant development, and sampling site affected the *T. orientalis* root-associated microbiome. Our results showed that the microbial community composition, diversity, and assembly processes of the root endosphere were significantly different from those found in water and soils (Fig. 1 to 3), and that microbiota diversity decreased in the root endosphere, suggesting that *T. orientalis* roots selectively recruit a subset of microbes from the environment. For example, many dominant bacterial classes (e.g., Gammaproteobacteria) and fungal classes (e.g., Dothideomycetes, Tremellomycetes, Eurotiomycetes, Leotiomycetes, Saccharomycetes, Microbotryomycetes, and Agaricomycetes) enriched in the root endosphere to certain degrees (Fig. 3a). This result is entirely consistent with previous findings, showing that plants harbored distinct microbiota, representing a subset of those found in the ambient environment (5, 10). It is generally assumed that roots are effective habitat filters, and root microbiome acquisition is a continuous process of gradual filtration (6, 28). Consequently, microbes first colonize the rhizosphere, and then invade the roots (9, 11). We speculated that the specific habitat characteristics of wetland root microenvironments might have significant influences on their microbial community. Previous studies have shown that hydrologic connectivity was a major factor in structuring microbial communities in most ecosystems (29, 30). Our source analysis also suggested that microbes in the rhizosphere and water were contributing microbial sources in the root endosphere (Fig. 3b). Generally, the assembly process of microbial communities in *T. orientalis* roots is believed to include enrichment from water and soil, then into the rhizosphere, and finally selectively colonizing in the root endosphere. However, due to the special habitats of wetlands, sampling methodologies might not allow the isolation of genuine rhizospheric microbes from the overwhelming majority of soil microorganisms, resulting in a partial representation of the rhizosphere microbes in our data (2, 31, 32). Indeed, we found that microbes in water and soils were more sensitive to sampling site than the root endosphere (Tables S4 and 5), where microbiomes remained relatively stable, potentially because of strong selection pressure from the host (33).

Plants modulate root microbiota primarily through root exudates and their immune system, and root microbiota expands the range and metabolic capacity of the plants, involving various processes such as nutrient requirement, plant development, and stress resistance (34–38). Therefore, plant development may drive the formation and alteration of the root-associated microbiome. Our results indicate that plant developmental stage has a greater impact on the root-associated microbial community than sampling site (Fig. 1 and Table S5). This result might be explained by the expectation that the plant microbiome changes over time, depending on abiotic and biotic environmental constraints (39–42). These results are consistent with previous studies that found plant development status significantly influences microbiomes in grass phyllosphere and *Arabidopsis* rhizosphere (16, 43, 44). The composition of root exudate is known to vary among plant species, developmental stages, and environments (45–47). During plant growth, nutritional and environmental conditions support plant reproductive growth at different stages. In this study, specific microbes were enriched in rhizosphere soil and root endosphere at different stages (Fig. S2), with some related to specific functions (11, 48). For example, bacterial classes Flavobacteria, Pseudomonadaceae, and Alcaligenaceae were enriched in rhizosphere soil and the root endosphere at the seedling stage, while Aeromonadaceae, Rhizobiaceae, and Enterobacteria were enriched in the root endosphere at the mature stage (Fig. S1 and2). However, the influences of plant developmental stage on the microbial communities include seasonal-related environmental factors, such as temperature. A Mantel analysis showed that temperature significantly affected bacterial and fungal communities (Table S6). Furthermore, environmental factors, such as ammonium nitrogen, total sulfur, phosphorus, sucrase, urease, and alkaline phosphatase also affected bacterial and fungal communities (Tables S6 and 8). Therefore, this study demonstrates the importance of wetland plant root-associated microbes in the carbon, nitrogen, and phosphorus cycles, reflecting the role of wetland plants in pollution control.

### Different responses of bacterial and fungal communities to plant development.

Plants provide niches for the growth, proliferation, and diversity of microbes, where bacteria and fungi are the most dominant forms. However, because bacteria and fungi differ greatly in size and lifestyle, we speculated that they might respond differently to the living environment. Firstly, the diversity of bacterial and fungal communities in the root endosphere responded differently at different developmental stages. The alpha diversity of the bacterial community in the root endosphere increased significantly with progressing plant developmental stage, while the alpha diversity of the fungal community did not differ significantly between the mature and wilting stage (Fig. 1 and Table S4). Further, a PERMANOVA analysis suggested that the structure of bacterial and fungal communities differed significantly at different developmental stages, where developmental stage explained 44.1% of bacterial community structure variation, and only 12.1% of fungal community structure variation within the root endosphere (Fig. 1 and Table S5). These results indicate that most bacteria respond much more rapidly to changes in environmental conditions than fungi (19).

Deterministic and stochastic assembly occurred concurrently along continuous chronosequences, and drove the spatial distribution of microbial communities (49, 50). Previous studies have shown that variability in distribution patterns of microbial communities may be related to the assembly process, and the response patterns of bacteria and fungi are different (51). Our results are consistent with this view, where the deterministic heterogeneous selection process dominated the assembly of bacterial communities in ecological processes, while dispersal limitation and stochastic drift processes dominated the assembly of fungal communities regardless of compartment niche (Fig. 2). It has previously been reported that bacterial community colonization of plants is not stochastic but controlled by specific assembly rules (9, 52, 53). Factors affecting the structure and composition of plant bacterial communities include soil type (10, 54), plant compartment (55, 56), host species (57, 58), plant immune system (59, 60), and plant developmental stage (43, 61). However, the establishment of fungal communities in the soil and plant is more susceptible to stochastic variations (62, 63), and responds differently to environmental factors (64, 65). Our results are consistent with previous studies that showed bacterial community assembly in the root endosphere was dominated by deterministic processes, and more susceptible to environmental factors such as temperature, sulfur content, and ammonium nitrogen. Moreover, they showed it was significantly associated with sucrase, urease, and alkaline phosphatase. In contrast, fungal community assembly was only significantly correlated with temperature (Tables S6 and 8). Langenheder et al. reported that the hydrological connectivity of the wetlands was a major factor affecting the assembly of microbial communities (66). It has been suggested that stochastic processes dominate microbial assembly in the aquatic environment, which may be reflected in the construction of fungal communities in the *T. orientalis* root endosphere (67). Bacterial colonization in the root endosphere was not a passive process, but under strong selection by the plant through important deterministic processes, perhaps reflecting the different sizes and lifestyles of bacteria and fungi.

These plant-related microbes can form complex associations, and have important roles in promoting the growth and health of plants (68, 69). Network analysis revealed that the interactions between bacteria and fungi changed with developmental stage (Fig. 4). The strongest interaction was observed among microbes at the mature stage, and the highest proportion of negative correlations also occurred at the mature stage, potentially due to the higher values of average degree and average closures centrality, and indicating that the network is more stable because competition improves microbial community stability (70, 71). However, an analysis of the network keystone taxa at each stage found them to almost cluster with bacteria at the seedling and mature stages, while fungi played more significant roles at the wilting stage. Further analyses of network keystones in the root endosphere microbiome revealed that bacterial keystones dominated in the bulk and rhizosphere soil environment, while fungal keystones were important at all growth stages (Table S11). These results indicate that plants likely create distinct niches for specific microbes, and can recognize their signal molecules, thus adapting their immune systems accordingly (72, 73). Plants might also selectively regulate microbial interactions to meet requirements during their growth, as microbial network keystones are expected to play vital roles in maintaining plant health and nutrient (24, 70).

### The ecological functions of bacterial and fungal communities, and keystone species in the root endosphere across plant developmental stages.

Our results demonstrate that the *T. orientalis* root endosphere is enriched for Gammaproteobacteria (Fig. 2). Previous studies have shown that Gammaproteobacteria members colonize plants, and play a key role in regulating host adaptability, pathogen inhibition, and plant stress tolerance (74, 75). In this study, the dominant bacterial taxa in the root endosphere varied significantly throughout plant development, with Pseudomonas and *Flavobacterium* enriched at the seedling stage (Fig. S2). Moreover, keystone taxa in the network indicated that the *Flavobacterium* and *Burkholderiales* OTUs had more connections with other taxa at the seedling stage (Table S11). The rhizobacteria Pseudomonas have previously been reported to facilitate plant growth (76–79). *Flavobacterium* also showed beneficial functions supporting plant growth and pathogen resistance (80, 81). Members of *Burkholderia* are important diazotrophs, and plant growth-promoting rhizobacteria (PGPR) (82, 83) with nitrogen-fixing and phosphorus solubilizing abilities (84, 85). These taxa have all been identified as important PGPR (86, 87) that can promote and improve host adaptability and performance through activities such as indoleacetic acid (IAA) production, siderophore production, and induced systemic resistance (88, 89). This finding was further supported by function prediction analysis of FAPROTAX, which indicated that function of chemoheterotrophy and aerobic chemoheterotrophy were significantly more abundant at earlier plant developmental stages to promote plant growth (Fig. 4b and Fig. S5). Moreover, metabolic processes associated with carbon and nitrogen became more abundant with progressive plant developmental stages (Fig. 4b and Fig. S5), indicating that bacterial communities play important ecological roles in maintaining plant health and nutrient requirements at earlier developmental stages.

Previous studies have suggested that most fungi colonizing plant tissues were mainly Ascomycetes and Basidiomycetes (48, 90–92), which is consistent with our results (Fig. 2). The majority of Ascomycetes are saprophytic fungi that decompose refractory organic matter, such as lignin and keratin, and play a crucial role in the nutrient cycle (93). Basidiomycetes can degrade plant residues, and play a decisive role in maintaining ecosystem balance and promoting material circulation (15, 94). Our results indicate that *Cladosporiaceae*, *Pleosporaceae*, and *Mortierellaceae* are sensitive to plant developmental stage, and are enriched at the seeding and wilting stage (Fig. S2). *Cladosporiaceae* has been reported to be highly resistant to environmental stress. *Pleosporaceae* is a common taxon of fungi with diverse lifestyles, including saprophytic, pathogenic, and endophytic. *Pleosporaceae* play different roles at different plant developmental stages, present as endophytic fungi in healthy plant tissues but as saprophytic fungi in necrotic tissues (95, 96). Some strains of *Mortierellaceae* are PGPF, and improve nutrient uptake and protect plants from pathogens (94). Some Dothideomycetes, Agaricomycetes, and Eurotiomycetes OTUs were key connections in the interaction network of the root endosphere. Previous studies have shown that fungal connectors belonging to Dothideomycete (97) and Agaricomycetes (98) have the potential to improve nutrient access, combat pathogenic taxa, and maintain cooperative metabolic associations with other species. This finding was further supported by function prediction analysis of FUNGuild, which indicated that function of Soil Saprotroph and Wood Saprotroph were significantly more abundant at later plant developmental stages. These results suggest that fungi were involved in nutrient acquisition in plant developmental stages, and play an important ecological role as decomposers at the wilting stage (99).

### Conclusion.

The microbial diversity and composition along the water-soil-plant continuum were related to their niche and the developmental stage of the plant, but the sampling site has little influence. As a plant barrier, roots selectively recruit specific microbial taxa primarily from water and rhizosphere soils. The assembly mechanisms of bacteria and fungi were different. While bacterial communities were dominated by deterministic processes, fungal communities were instead driven by stochastic processes. The effect of plant developmental stage on the bacterial community was greater than that on the fungal community, and the bacterial community was more vulnerable to biological and abiotic factors. The keystone taxa and ecological role of bacterial and fungal communities in the roots shifted significantly with plant development. Bacteria play an important role in maintaining plant health and nutrient requirements at the early stages, while fungi take an increasingly greater role as the decomposer at later stages. These findings significantly advance our understanding of the assembly, function, and interactions of the plant microbiome in the wetland ecosystem, and provide fundamental knowledge for future synthetic community research.

## MATERIALS AND METHODS

### Study area, sample collection, and environmental properties.

The sampling area (112°23′3″E, 37°36′28″N) is located in the river wetland with surface runoff flowing into the Fenhe River, the largest tributary of the Yellow River, in Taiyuan, Shanxi Province, China, where *T. orientalis* is the dominant aquatic macrophyte. We collected water, bulk soil, rhizosphere soil, and root samples at the seedling stage (April), mature stage (June), and wilting stage (October) of *T. orientalis* in 2021. Based on the data from investigations on the physical and chemical properties of water, 3 sample sites for inlet, middle, and outlet were set up across the surveyed wetland. At each sampling site (5 m × 5 m), 5 locations were sampled. For the water samples, 1.5 L of water was collected into a sterile plastic bucket, and filtered through a 0.2 μm pore size membrane filter (Millipore). Bulk soil samples were collected near the water samples. Rhizosphere soil (defined as soil tightly attached to the roots) of the plant was collected afterward. At each sampling site, 1 to 2 roots from each of 5 plants were clipped and immediately placed into an ice bag. To sample the root endosphere microbiome, the roots were surface-sterilized for 2 min in 70% ethanol and subsequently sterilized again for 5 min in 0.25% NaClO (0.1% Tween 80), then sterilized for 30 sec in 70% ethanol, and washed with sterile water 3 times. In total, we collected 162 samples for microbial community analysis. All samples were transported to the laboratory on dry ice and stored at −80°C until DNA extraction.

The soil samples were stored at 4°C for physicochemical analysis. Soil TC and total nitrogen (TN) were quantified by elemental analysis (Vario MACRO; Elementar). SOC was measured using the K_2_Cr_2_O_7_ oxidation method. Available phosphorus (AP) was measured using the NaHCO_3_ extraction-colorimetric method. Soil pH was quantified in a 1 M KCl soil suspension with a soil:water ratio of 1:2.5 (wt/vol) (Hanna Instruments). Ammonium nitrogen and nitrate nitrogen (NO_3_^−^-N) were quantified by automated discrete analysis (CleverChem 380; DeChem-Tech. GmbH). Water physical parameters, including Temp, pH, DO, EC, nitrate (NO_3_-), and ammonium (NH_4_^+^) content, were measured *in situ* using a portable water multiparameter quality monitor (AP-2000; Aquaread). Total TP was quantified by automated discrete analysis (CleverChem 380). Soil sucrase was measured by 3,5-dinitrosalicylic acid colorimetry. Urease was measured by sodium phenol sodium hypochlorite colorimetry. Alkaline phosphatase was measured by phenylene phosphate disodium colorimetry. All measured parameters are listed in Tables S1 to 3.

### DNA extraction.

Filters with retained biomass were cut into pieces and placed into centrifuge tubes. The roots were pulverized with a mortar and pestle in liquid nitrogen. DNA was extracted from all biomass, root, rhizosphere, bulk soil, and root endosphere samples using the FastDNA Spin Kit (MP Biomedicals Inc) according to the manufacturer’s protocol.

### Illumina sequencing analysis.

We amplified the V5 to V6 region of the bacterial 16S rRNA (rRNA) gene using primers 799F (5′-AACMGGATTAGATACCCKG-3′) and 1115R (5′-ACGTCATCCCCACCTTCC-3′), and the fungal ITS2 region using primers ITS1F (5′-CTTGGTCATTTAGAGGAAGTAA-3′) and ITS2R (5′-GCTGCGTTCTTCATCGATGC-3′), and the following thermal cycling parameters: initial denaturation at 95°C for 3 min, followed by 27 cycles of denaturing at 95°C for 30 s, annealing at 55°C for 30 s, and extension at 72°C for 45 s, and single extension at 72°C for 10 min before being held at 10°C. The PCR mix contained 4 μL 5× *TransStart* FastPfu buffer, 2 μL 2.5 mM dNTPs, 0.8 μL forward primer (5 μM), 0.8 μL reverse primer (5 μM), 0.4 μL *TransStart* FastPfu DNA polymerase, 10 ng DNA, and double-distilled water (ddH_2_O) for a final volume of 20 μL (TransGen Biotech). All PCRs were performed in triplicate. PCR products were extracted from a 2% agarose gel, and purified using the AxyPrep DNA Gel Extraction Kit (Axygen Biosciences) according to the manufacturer’s recommended protocol, and quantified using a Quantus Fluorometer (Promega).

Purified PCR amplicons were pooled in equimolar amounts, and paired-end sequenced on either an Illumina MiSeq PE300 or NovaSeq PE250 platform (Illumina) according to standard protocols by Majorbio Bio-Pharm Technology Co. Ltd.

Operational taxonomic units (OTUs) were clustered with a 97% similarity cutoff using UPARSE v.7.1, and chimeric sequences were identified and removed. The taxonomy of each OTU representative sequence was analyzed by RDP Classifier v.2.2 against the 16S rRNA internal transcribed spacer (ITS) database, using a confidence threshold of 0.7. Bacterial functional profiles were predicted using PICRUSt2 and functional annotation of prokaryotic taxa (FAPROTAX). The bacterial OTUs were compared with the data set obtained by FAPROTAX (script version 1.1), and the output functional table used the default settings. Fungal functional profiles were inferred using PICRUSt2, and the program FUNGuild, where FUNGuild v1.0 was used to determine the functional group of fungi. FUNGuild v1.0 is a flat database hosted by GitHub (https://github.com/UMNFuN/FUNGuild).

### Statistical analysis.

A one-way analysis of variance (ANOVA) was used to assess the significance of differences in the environmental parameters, alpha diversity indices, and the relative abundance of dominant microbial classes. Microbial beta diversity was assessed by first computing Bray-Curtis dissimilarity matrices, and then ordinating using NMDS. The significance of different factors on community dissimilarity was assessed with permutational multivariate analysis of variance (PERMANOVA) or Nested PERMANOVA using the ‘adonis’ function of the vegan package in the R statistical software.

To assess the relative importance of deterministic and stochastic processes in microbiome assembly, we calculated the beta Nearest Taxon Index βNTI using a null model, and defined |βNTI| ≥ 2 as dominant deterministic processes and |βNTI| < 2 as dominant stochastic processes (29, 100). Further, deterministic and stochastic processes were partitioned into 5 ecological processes based on both βNTI and the Bray-Curtis-based Raup-Crick Index (RC_Bray_) values: heterogeneous selection (βNTI < -2), homogeneous selection (βNTI > 2), dispersal limitation (|βNTI| < 2 and RC_Bray_ > 0.95), homogenizing dispersal (|βNTI| < 2 and RC_Bray_ < -0.95), and undominated (|βNTI| < 2 and |RC_Bray_| < 0.95) (20, 29).

The co-occurrence network was constructed with the WGCNA package in R. Spearman’s rank correlation scores (*r*) were calculated, and only robust (*r *>* *0.6 or *r* < -0.6) and statistically significant (*P* < 0.01) correlations were retained. We used Source Tracker (v.1.0) based on a Bayesian approach to estimate the sources of the microbial communities. The networks were visualized in Gephi (101). A Mantel test was performed to explore Spearman’s rank correlations between microbial communities, soil physicochemical characteristics, and soil enzyme activities using the vegan package in R. Statistical analyses were performed using software SPSS 20.0 (IBM SPSS statistics) and R (http://www.r-project.org).

### Data availability.

All sequence data were deposited in the NCBI SRA database under BioProject ID PRJNA839557 and PRJNA839553.
