# Supplementary material for: Ecological Processes of Bacterial and Fungal Communities Associated with Typha orientalis Roots in Wetlands Were Distinct during Plant Development
Source: Microbiol Spectr. 2023 Jan 23;11(1):e05051-22. doi: 10.1128/spectrum.05051-22 (PMC9927475; doi:10.1128/spectrum.05051-22)
Supplement: Supplemental file 1 — Supplemental material. Download spectrum.05051-22-s0001.pdf, PDF file, 2.2 MB [file spectrum.05051-22-s0001.pdf]

Supplementary Table 1 **The soil properties at three developmental stages in three sampling sites**

stage	Samples	Site	N（%）	C（%）	S（%）	pH	NH_4^+-N（mg/l）	NO_3^--N（mg/l）	TP（mg/kg）	SOC(%)
Seedling stage	Bulk soil	Int	0.025±0.001a	1.251±0.013a	0.083±0.001a	7.890±0.030ab	0.528±0.099a	0.141±0.011a	2.600±0.279a	0.598±0.042a
		Mid	0.025±0.002a	1.256±0.062a	0.060±0.010b	8.064±0.034a	0.393±0.025ab	0.109±0.024ab	3.550±0.800a	0.470±0.103a
		Out	0.035±0.008a	1.455±0.215a	0.044±0.001b	8.032±0.067ab	0.318±0.045b	0.078±0.007b	4.325±0.531a	0.510±0.155a
	Rhizosphere	Int	0.016±0.001a	1.022±0.016	0.118±0.012a	8.268±0.049a	0.329±0.046a	0.050±0.010a	4.375±0.439a	0.3575±0.0386
		Mid	0.013±0.000a	0.938±0.015a	0.079±0.013ab	8.212±0.024a	0.253±0.017a	0.049±0.006a	5.825±1.97a	0.223±0.019a
		Out	0.022±0.006a	1.197±0.179a	0.065±0.013b	8.034±0.042b	0.123±0.030b	0.051±0.014a	4.775±0.579a	0.335±0.139a
Mature stage	Bulk soil	Int	0.034±0.008a	1.457±0.247a	0.044±0.008a	7.94±0.597a	0.611±0.026a	0.034±0.005a	4.225±0.657a	0.598±0.042a
		Mid	0.027±0.006a	1.272±0.180a	0.041±0.002a	8.48±0.098a	0.615±0.048a	0.032±0.001a	3.975±0.442a	0.470±0.103a
		Out	0.032±0.009a	1.396±0.297a	0.025±0.003b	8.778±0.037a	0.444±0.050b	0.033±0.001a	6.025±0.753a	0.510±0.155a
	Rhizosphere	Int	0.052±0.008a	1.617±0.221a	0.007±0.007a	8.312±0.023b	0.610±0.015a	0.038±0.003a	4.775±0.477a	0.690±0.112a
		Mid	0.021±0.004b	1.017±0.037b	0.049±0.004a	8.394±0.055ab	0.570±0.014ab	0.028±0.002a	3.800±0.986a	0.290±0.041b
		Out	0.024±0.003b	1.037±0.065b	0.028±0.004b	8.508±0.034a	0.487±0.050b	0.034±0.004a	4.55±0.393a	0.297±0.036b
Wilting stage	Bulk soil	Int	0.023±0.003a	1.297±0.169a	0.091±0.012a	7.970±0.110b	0.421±0.046a	0.094±0.003a	2.975±0.232b	0.447±0.069a
		Mid	0.018±0.002a	0.974±0.050ab	0.055±0.003b	8.183±0.038ab	0.297±0.005b	0.058±0.002b	4.850±0.253a	0.202±0.027b
		Out	0.018±0.003a	0.912±0.042b	0.046±0.002b	8.377±0.038a	0.272±0.022b	0.029±0.016b	3.750±0.719ab	0.247±0.057b
	Rhizosphere	Int	0.017±0.002a	1.006±0.021a	0.045±0.001a	8.053±0.145a	0.378±0.046a	0.091±0.042a	3.800±0.594a	0.332±0.089a
		Mid	0.013±0.001a	0.956±0.015a	0.038±0.002b	8.187±0.050a	0.277±0.036a	0.069±0.012b	3.000±0.147a	0.240±0.020a
		Out	0.016±0.001a	0.939±0.056a	0.033±0.001c	8.360±0.110a	0.318±0.017a	0.056±0.019b	4.550±0.661a	0.282±0.059a

The data represents mean ± SD. Different letters alongside the number indicate a significant difference determined by Duncan test on ANOVA.

Supplementary Table 2 **The water properties at three developmental stages in three sampling sites**

stage	Site	Temp (℃)	pH	DO (mg/L)	EC (uS/cm)	TP（mg/L）	NO_3^--N（mg/l）	NH_4^+-N（mg/l）
Seedling stage	Int	14.700±0.057b	7.810±0.002b	9.440±0.046a	2022.7±11.2a	0.440 ±0.115b	2.753±0.049a	0.183±0.003b
	Mid	14.867±0.120ab	7.560±0.003c	7.900±0.122b	1944.0±7.6b	0.510±0.095b	2.673±0.085a	0.133±0.003c
	Out	15.067±0.033a	7.893±0.007a	9.626±0.441a	1882.0±5.0c	0.887±0.078a	2.447±0.112a	0.263±0.013a
Mature stage	Int	24.867±0.067a	7.433±0.003a	3.690±0.052a	2359.0±2.0a	0.300±0.104b	6.153±0.039a	0.510±0.021a
	Mid	23.967±0.033b	7.373±0.009b	2.237±0.088b	2344.3±6.2a	1.980±0.078a	5.793±0.032b	0.437±0.009b
	Out	23.967±0.033b	7.363±0.003b	2.44±0.038b	2305.0±4.0b	0.850±0.319b	4.963±0.055c	0.460±0.012ab
Wilting stage	Int	9.467±0.133a	7.887±0.026a	4.857±0.156a	922.0±10.5a	1.293±0.533a	5.703±0.632b	0.247±0.049a
	Mid	8.900±0.115b	7.813±0.033a	3.557±0.446b	877.7±0.9b	1.460±0.866a	6.103±0.096b	0.317±0.009a
	Out	8.867±0.033b	7.800±0.021a	4.840±0.342a	796.7±3.7c	1.123±0.493a	10.563±0.024a	0.287±0.003a

The data represents mean ± SD. Different letters alongside the number indicate a significant difference determined by Duncan test on ANOVA.

Supplementary Table 3 **The Soil enzyme activity at three developmental stages in three sampling sites**

stage	Samples	Site	Urease (IU/g)	Sucrase (IU/g)	Alkaline phosphatase (IU/g)
Seedling stage	Bulk soil	Int	63.804±0.671a	156.027±1.627a	11.335±0.123a
		Mid	58.141±0.756b	150.537±0.960b	10.820±0.100b
		Out	56.291±0.925b	145.993±1.626b	10.372±0.151c
	Rhizosphere	Int	65.729±0.495a	130.544±1.136a	11.403±0.118a
		Mid	65.087±0.709a	126.005±1.486b	11.163±0.109ab
		Out	65.389±0.424a	123.696±0.353b	10.795±0.120b
Mature stage	Bulk soil	Int	60.157±0.544a	127.578±0.125a	11.078±0.099a
		Mid	59.499±0.284a	126.738±1.299a	11.286±0.217a
		Out	57.229±0.830b	125.451±0.402a	10.989±0.066a
	Rhizosphere	Int	62.271±0.459a	155.660±1.482a	13.268±0.105a
		Mid	61.667±0.633a	140.817±3.616b	13.081±0.097ab
		Out	61.727±0.916a	133.490±3.212b	12.899±0.0923b
Wilting stage	Bulk soil	Int	61.216±0.472a	160.623±1.889a	13.465±0.074a
		Mid	60.271±0.239ab	158.634±1.528a	12.370±0.074b
		Out	60.011±0.189b	152.790±0.762b	12.163±0.041b
	Rhizosphere	Int	71.170±0.216a	156.122±1.364a	13.288±0.090a
		Mid	69.490±0.408b	152.881±1.303a	12.365±0.066b
		Out	68.135±0.050c	148.481±0.933b	12.567±0.108b

The data represents mean ± SD. Different letters alongside the number indicate a significant difference determined by Duncan test on ANOVA.

Supplementary Table 4 **Effects of multiple biotic and abiotic factors on microbial alpha diversity**

Samples	Variables	Bacteria			Fungi		
		F value	df	*P*	*F* value	df	*P*
All samples (n = 162)	Compartment niche	229.590	3	**< 0.001**	137.771	3	**< 0.001**
	Developmental stage	10.484	2	**< 0.001**	9.724	2	**< 0.001**
	Site	1.675	2	0.191	1.131	2	0.326
Water (n = 27)	Developmental stage	36.718	2	**< 0.001**	230.863	2	**< 0.001**
	Site	3.404	2	0.056	16.810	2	**< 0.001**
Bulk soil (n = 45)	Developmental stage	2.743	2	0.078	9.111	2	**0.001**
	Site	2.785	2	0.075	1.094	2	0.345
Soil rhizosphere (n = 45)	Developmental stage	0.289	2	0.750	2.042	2	0.143
	Site	3.148	2	0.055	0.373	2	0.691
Root endosphere (n = 45)	Developmental stage	54.654	2	**< 0.001**	5.437	2	**< 0.001**
	Site	0.186	2	0.931	3.539	2	**0.008**

The effects of multiple factors on microbial alpha diversity was tested with Multi-way ANOVA analysis based on Chao1 richness. Relative contributions of interactions between the multiple factors are not shown.

Supplementary Table 5 **Effects of multiple biotic and abiotic factors on the microbiome assembly**

Samples	Variables	Bacteria		Fungi	
		R^2	*P*	R^2	*P*
All samples (n = 162)	Compartment niche	0.379	**0.001**	0.172	**0.001**
	Developmental stage	0.078	**0.001**	0.066	**0.001**
	Site	0.013	0.327	0.027	**0.001**
Water (n = 27)	Developmental stage	0.775	**0.001**	0.693	**0.001**
	Site	0.057	0.595	0.090	0.325
Bulk soil (n = 45)	Developmental stage	0.189	**0.001**	0.172	**0.001**
	Site	0.086	**0.005**	0.151	**0.001**
Soil rhizosphere (n = 45)	Developmental stage	0.135	**0.001**	0.119	**0.001**
	Site	0.082	**0.006**	0.072	**0.004**
Root endosphere (n = 45)	Developmental stage	0.441	**0.001**	0.121	**0.001**
	Site	0.038	0.663	0.048	0.299

The significance of different factors on microbial community dissimilarity was tested with PERMANOVA (based on bray-curits distances). Relative contributions of interactions between the multiple factors are not shown.

Supplementary Table 6 **Spearman's correlations between microbial communities and soil physicochemical characteristics based on Mantel tests**

		Bcteria			Fungi		
		Bulk soil	Rhizosphere soil	Root endosphere	Bulk soil	Soil rhizosphere	Root endosphere
Temp (℃)	r	0.181	0.179	0.37	0.177	0.085	0.084
	P	**0.001**	**0.001**	**0.001**	**0.001**	**0.043**	**0.036**
N（%）	r	0.022	0.065	0.018	-0.055	-0.026	0.097
	P	0.81	0.525	0.723	0.541	0.801	0.239
C（%）	r	0.024	0.022	0.05	-0.058	0.011	0.033
	P	0.768	0.861	0.39	0.452	0.928	0.716
S（%）	r	-0.115	-0.036	0.245	0.092	0.053	0.079
	P	0.159	0.646	**0.001**	0.241	0.443	0.189
pH	r	-0.124	0.049	0.049	-0.106	-0.062	-0.06
	P	0.162	0.627	0.348	0.311	0.477	0.424
NH_4^+-N（mg/l）	r	0.161	0.148	0.211	0.064	0.281	0.042
	P	**0.048**	0.104	**0.001**	0.437	**0.006**	0.582
NO_3^--N（mg/l）	r	0.064	-0.145	0.074	0.07	-0.059	0.032
	P	0.374	0.132	0.174	0.328	0.548	0.722
TP（mg/kg）	r	0.233	0.053	0.075	0.091	0.111	0.063
	P	**0.007**	0.524	0.116	0.258	0.159	0.396
SOC(%)	r	0.005	-0.001	0.048	0.146	0.051	-0.078
	P	0.953	0.924	0.32	**0.038**	0.519	0.246

Supplementary Table 7 **Spearman's correlations between microbial communities and water physicochemical characteristics based on Mantel tests**

		Bacteria	Fungi
Temp (℃)	r	0.311	0.753
	P	**0.001**	**0.001**
pH	r	0.113	0.456
	P	**0.039**	**0.001**
DO (mg/L)	r	0.374	0.628
	P	**0.001**	**0.001**
EC (uS/cm)	r	0.285	0.68
	P	**0.002**	**0.001**
TP（mg/L）	r	0.081	0.088
	P	0.278	0.055
NO_3^--N（mg/l）	r	0.485	0.626
	P	**0.001**	**0.001**
NH_4^+-N（mg/l）	r	0.46	0.448
	P	**0.001**	**0.001**

Supplementary Table 8 **Spearman’s correlations between microbial communities and soil enzyme activities based on Mantel tests**

		Bcteria			Fungi		
		Bulk soil	Rhizosphere soil	Root endosphere	Bulk soil	Rhizosphere soil	Root endosphere
Sucrase	r	-0.038	0.190	0.456	0.015	0.095	0.009
	P	0.490	**0.001**	**0.001**	0.773	**0.023**	0.806
Urease	r	0.017	0.177	0.182	0.105	-0.059	-0.025
	P	0.853	**0.007**	**0.002**	0.259	0.394	0.682
ALP	r	0.089	0.136	0.572	0.310	0.160	0.034
	P	0.267	**0.046**	**0.001**	**0.001**	**0.021**	0.538

Supplementary Table 9 **The parameters of microbial interaction co-occurrence networks**

	**Node**	**edge**	**Bacteria node**	**Fungi node**	**Average degree**	**Bacteria degree**	**Fungi degree**	**Modularity**	**Average clustering coefficient**	**Average path distance**	**network density**	**Postive edge**	**Negative edge**
Seeding stage	266	8602	64 (24.06%)	202 (75.94%)	64.677	79.25	60.06	0.104	0.849	1.574	0.244	93.66%	6.34%
Mature stage	319	11559	96 (30.09%)	223 (69.91%)	72.47	105.59	58.21	0.127	0.797	1.71	0.228	73.95%	26.05%
Wilting stage	298	2076	88 (29.53%)	210 (70.47%)	13.933	23.57	14.52	0.32	0.611	2.46	0.047	83.19%	16.81%

Supplementary Table 10 **The parameters of microbial interaction co-occurrence networks for each niche**

		Node	edge	Bacteria node	Fungi node	Average degree	Bacteria degree	Fungi degree	Modularity	Average clustering coefficient	Average path distance	network density	Postive edge	Negative edge
Water	Seeding stage	173	2081	51 (29.48%)	122 (70.52%)	24.058	36.25	63.80	0.309	0.216	2.154	0.14	56.64%	45.36%
	Mature stage	192	3517	38 (19.79%)	154 (80.21%)	36.635	33.50	37.86	0.325	0.35	2.002	0.192	50.36%	49.64%
	Wilting stage	160	2319	32 (20.0%)	128 (80.0%)	28.988	28.69	29.81	0.338	0.345	2.123	0.182	63.86%	36.14%
Bulk soil	Seeding stage	268	1891	82 (30.6%)	186 (69.4%)	14.112	16.54	13.04	0.475	0.202	2.678	53	79.69%	20.31%
	Mature stage	272	2052	91 (33.46%)	181 (66.54%)	15.088	17.20	14.03	0.477	0.232	2.678	0.056	75.93%	24.07%
	Wilting stage	287	1274	97 (33.8%)	190 (66.2%)	8.878	10.07	8.27	0.607	0.181	3.43	0.031	92.70%	7.30%
Rhizosphere soil	Seeding stage	265	1448	84 (31.7%)	181 (68.3%)	10.928	14.07	9.47	0.475	152	3.099	0.041	82.67%	17.33%
	Mature stage	285	1203	112 (39.3%)	173 (60.7%)	8.442	8.81	8.20	0.634	0.153	3.465	0.03	96.67%	3.33%
	Wilting stage	292	979	85 (29.11%)	207 (70.89%)	6.705	7.31	6.46	0.69	0.12	3.982	0.023	98.37%	1.63%
Root endosphere	Seeding stage	181	176	32 (17.68%)	149 (82.32%)	1.945	2.59	1.81	0.774	0.006	6.837	0.011	100%	0
	Mature stage	246	58	64 (26.02%)	182 (73.98%)	4.618	5.20	4.41	0.705	0.129	4.717	0.019	99.65%	0.35%
	Wilting stage	294	767	86 (29.25%)	208 (70.75%)	5.218	19.23	21.21	0.714	0.121	4.307	0.018	98.04%	1.96%

Supplementary Table 11 **The keystones of microbial interaction co-occurrence networks**

	Label	taxa	phylum	class	family	degree
seeding stage	BOTU4652	Bacteria	Proteobacteria	Alphaproteobacteria	Rhizobiales_Incertae_Sedis	146
	BOTU5597	Bacteria	Actinobacteriota	Actinobacteria	Intrasporangiaceae	145
	FOTU4603	Fungi	Rozellomycota	unclassified_p__Rozellomycota	unclassified_p__Rozellomycota	144
	BOTU6765	Bacteria	Verrucomicrobiota	Verrucomicrobiae	Rubritaleaceae	143
	BOTU2592	Bacteria	Proteobacteria	Gammaproteobacteria	Comamonadaceae	143
	BOTU8371	Bacteria	Desulfobacterota	Desulfobulbia	Desulfocapsaceae	143
	BOTU5323	Bacteria	Proteobacteria	Gammaproteobacteria	SC-I-84	143
mature stage	BOTU2839	Bacteria	Proteobacteria	Gammaproteobacteria	Pectobacteriaceae	176
	BOTU2734	Bacteria	Proteobacteria	Gammaproteobacteria	Alteromonadaceae	173
	BOTU4715	Bacteria	Desulfobacterota	Desulfobulbia	Desulfocapsaceae	172
	BOTU2592	Bacteria	Proteobacteria	Gammaproteobacteria	Comamonadaceae	171
	BOTU4988	Bacteria	Actinobacteriota	Thermoleophilia	norank_o__Gaiellales	171
	BOTU5376	Bacteria	Proteobacteria	Gammaproteobacteria	Steroidobacteraceae	171
wilting stage	FOTU702	Fungi	unclassified_k__Fungi	unclassified_k__Fungi	unclassified_k__Fungi	83
	FOTU10463	Fungi	unclassified_k__Fungi	unclassified_k__Fungi	unclassified_k__Fungi	83
	BOTU5376	Bacteria	Proteobacteria	Gammaproteobacteria	Steroidobacteraceae	81
	FOTU4603	Fungi	Rozellomycota	unclassified_p__Rozellomycota	unclassified_p__Rozellomycota	75
	BOTU5432	Bacteria	Proteobacteria	Gammaproteobacteria	Nitrosomonadaceae	65
	FOTU2067	Fungi	unclassified_k__Fungi	unclassified_k__Fungi	unclassified_k__Fungi	65

Supplementary Table 12 **The keystones of root endosphere microbial interaction co-occurrence networks**

	Label	taxa	phylum	class	family	degree
seeding stage	FOTU5638	Fungi	Ascomycota	Eurotiomycetes	Aspergillaceae	20
	BOTU10779	Bacteria	Bacteroidota	Bacteroidia	Flavobacteriaceae	19
	FOTU5542	Fungi	Ascomycota	Saccharomycetes	Saccharomycetales_fam_Incertae_sedis	14
	FOTU8326	Fungi	Ascomycota	Sordariomycetes	Nectriaceae	14
	BOTU11250	Bacteria	Bacteroidota	Bacteroidia	Flavobacteriaceae	11
	BOTU6790	Bacteria	Proteobacteria	Gammaproteobacteria	Comamonadaceae	11
mature stage	FOTU7096	Fungi	Basidiomycota	Agaricomycetes	Peniophoraceae	34
	FOTU8643	Fungi	Ascomycota	Eurotiomycetes	Trichocomaceae	26
	FOTU3864	Fungi	Basidiomycota	Tremellomycetes	Filobasidiaceae	25
	BOTU2891	Bacteria	Proteobacteria	Gammaproteobacteria	Pectobacteriaceae	24
	FOTU5540	Fungi	Ascomycota	Dothideomycetes	Cladosporiaceae	23
	FOTU3833	Fungi	Basidiomycota	unclassified_p__Basidiomycota	unclassified_p__Basidiomycota	23
wilting stage	BOTU9883	Bacteria	Actinobacteriota	Actinobacteria	Microbacteriaceae	40
	BOTU11169	Bacteria	Firmicutes	Clostridia	Clostridiaceae	37
	FOTU2386	Fungi	Chytridiomycota	unclassified_p__Chytridiomycota	unclassified_p__Chytridiomycota	36
	FOTU7542	Fungi	unclassified_k__Fungi	unclassified_k__Fungi	unclassified_k__Fungi	36
	FOTU8730	Fungi	Ascomycota	Dothideomycetes	Didymellaceae	34

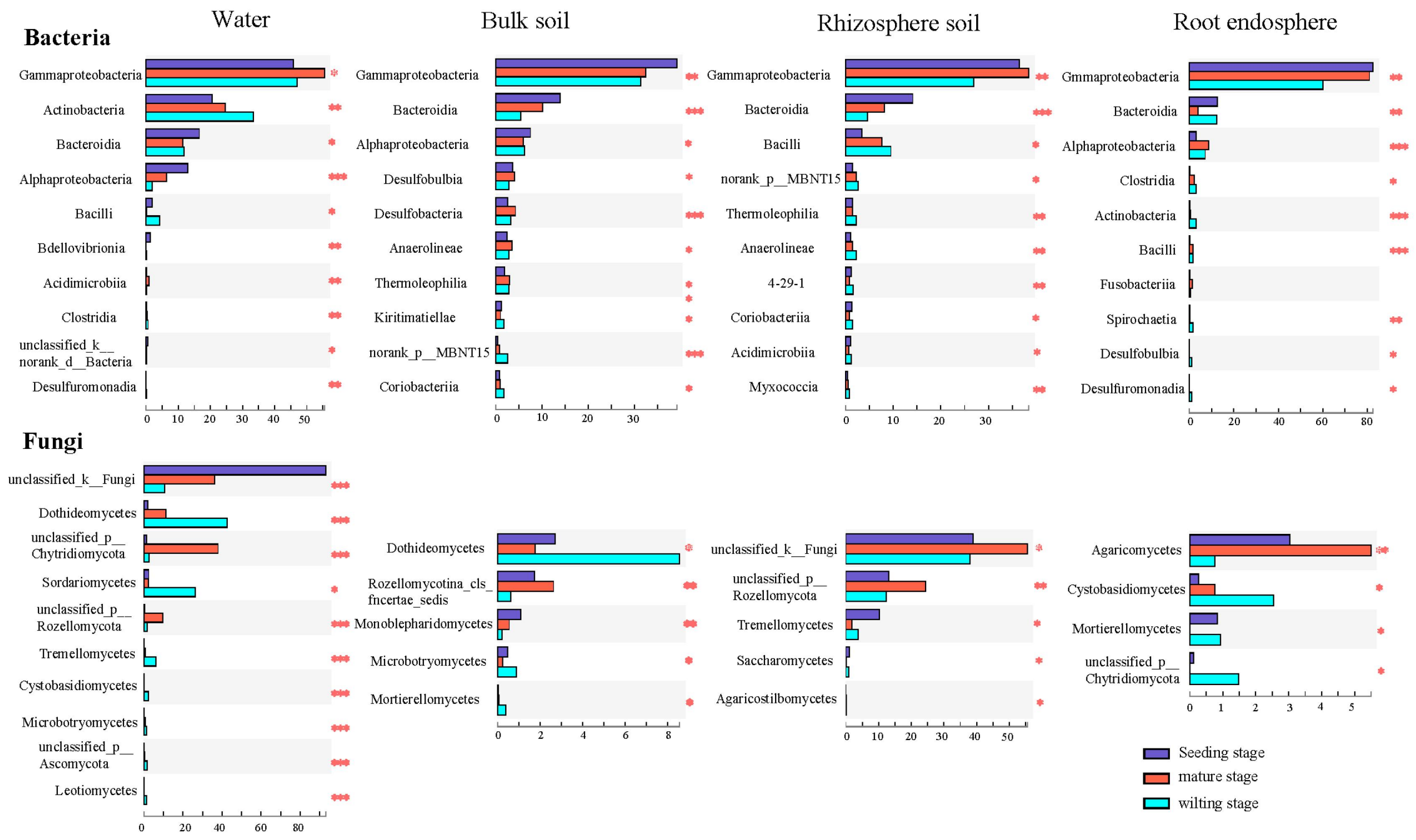

Supplementary Fig. 1 The variation analysis of dominant classes of bacterial and fungal communities in water, bulk soil, rhizosphere soil and root endosphere across plant developmental stages.

* indicates significant differences at the 0.05 level; ** indicates significant differences at the 0.01 level.

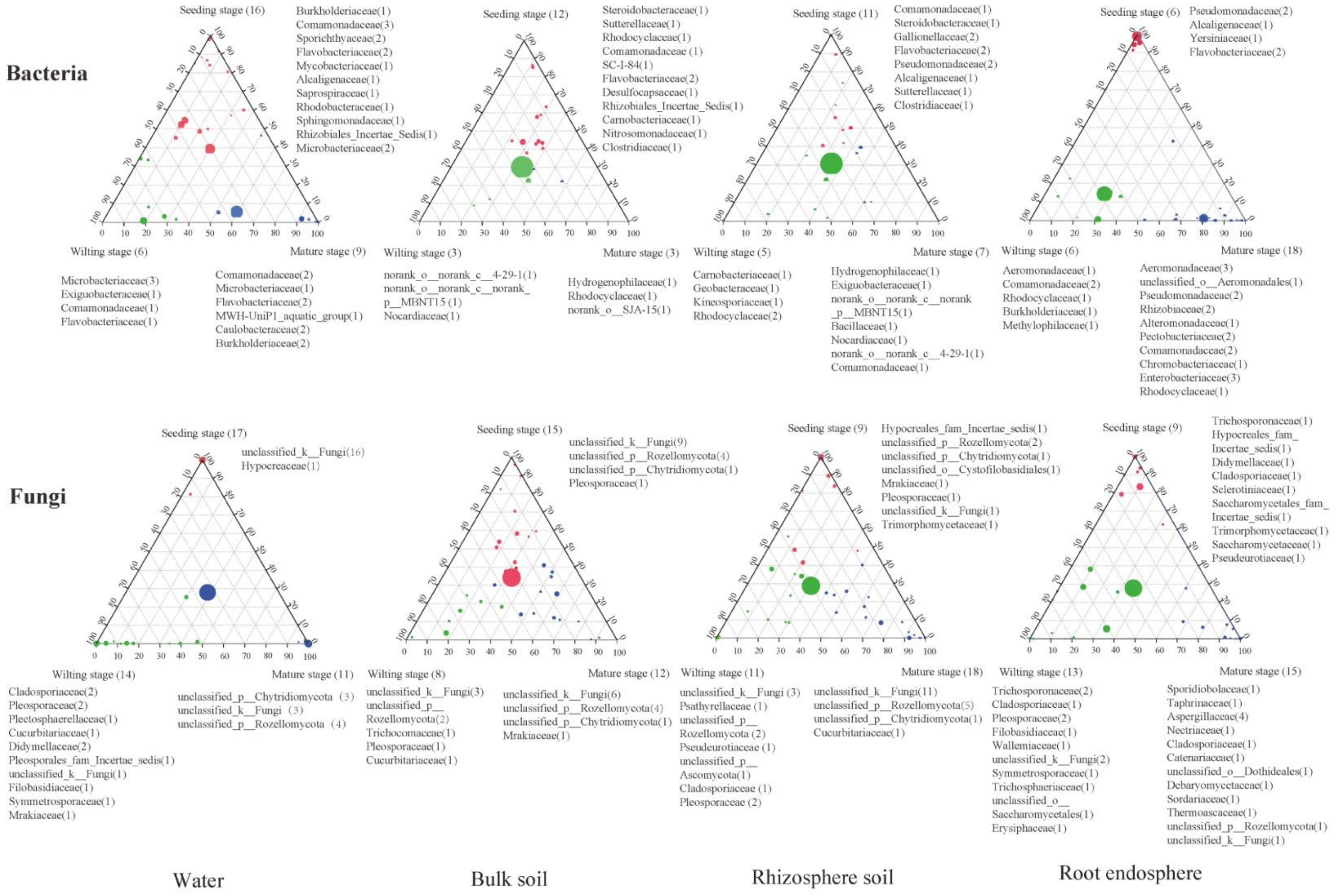

Supplementary Fig. 2 Ternary plots depicting bacterial and fungal OTUs significantly enriched in three developmental stages within each compartment niche (P < 0.01). Each circle represents one OTU, and the size of each circle represents its relative abundance.

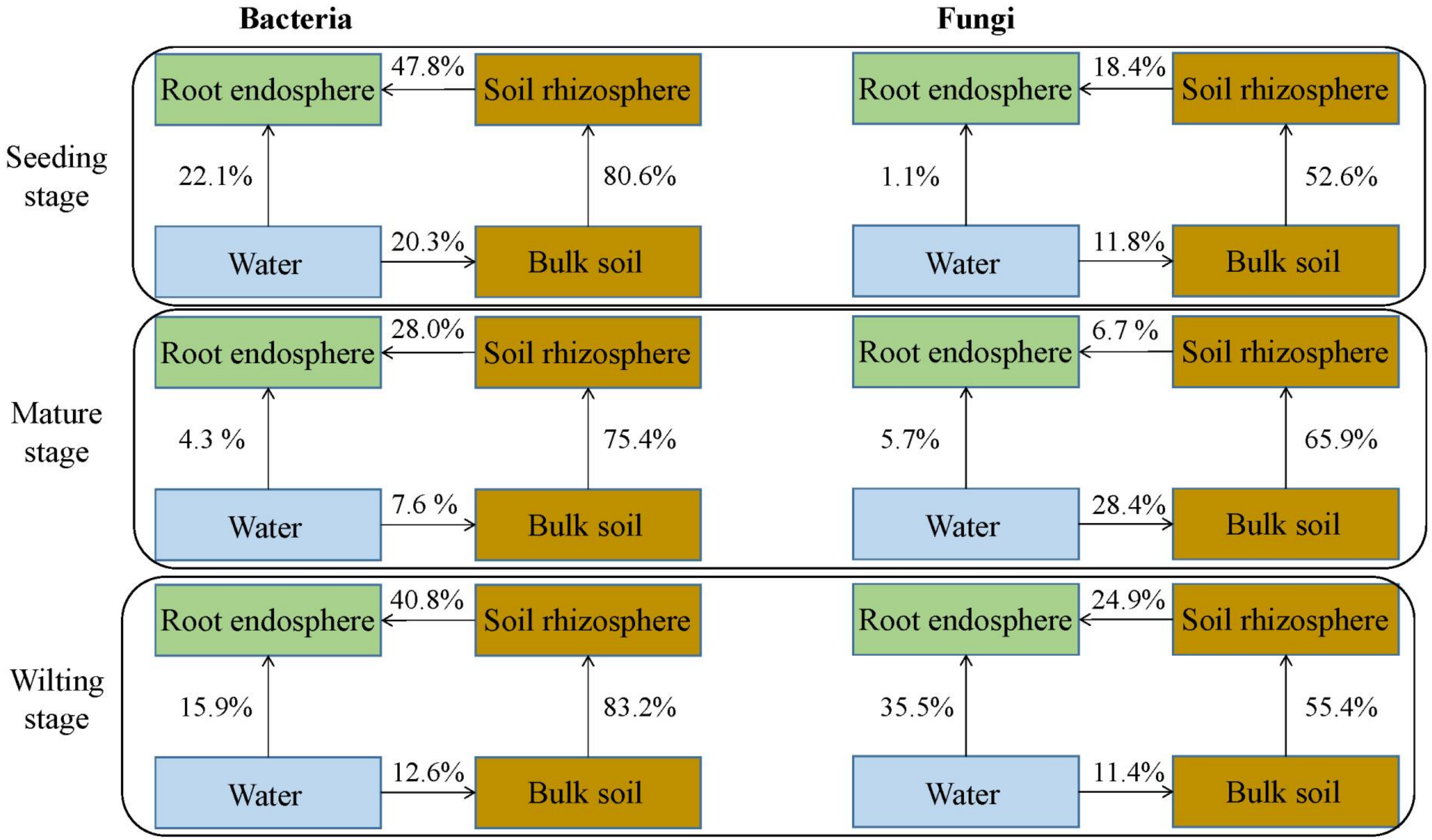

Supplementary Fig. 3 Potential source and contribution of root-associated bacterial and fungal communities in each plant developmental stage.

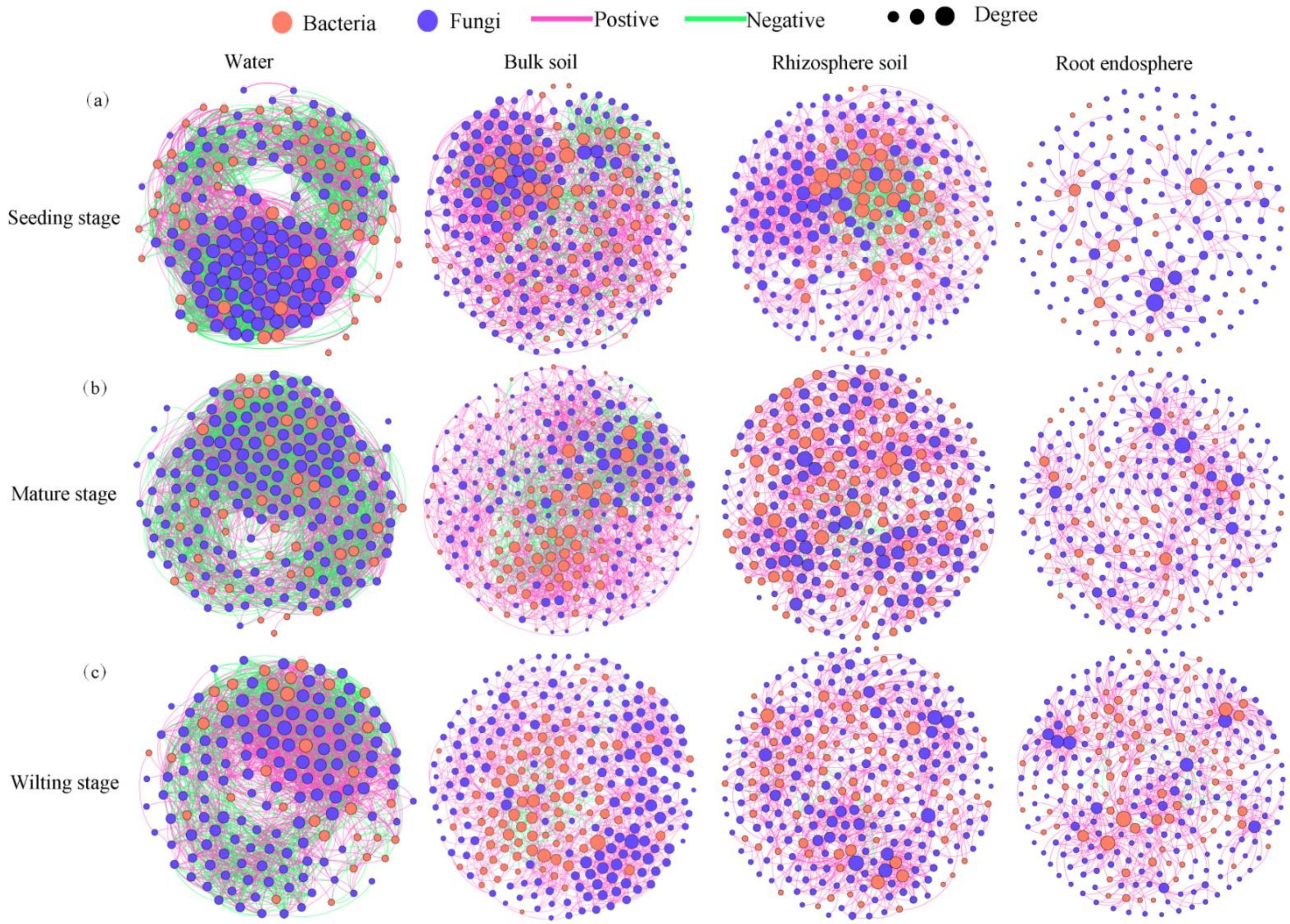

Supplementary Fig. 4 Temporal dynamics of microbial interaction networks within each niche. Co-occurrence network analysis of water, bulk soil, rhizoshere soil, root endosphere at (a) seeding stage, (b) mature stage, (c) wilting stage.

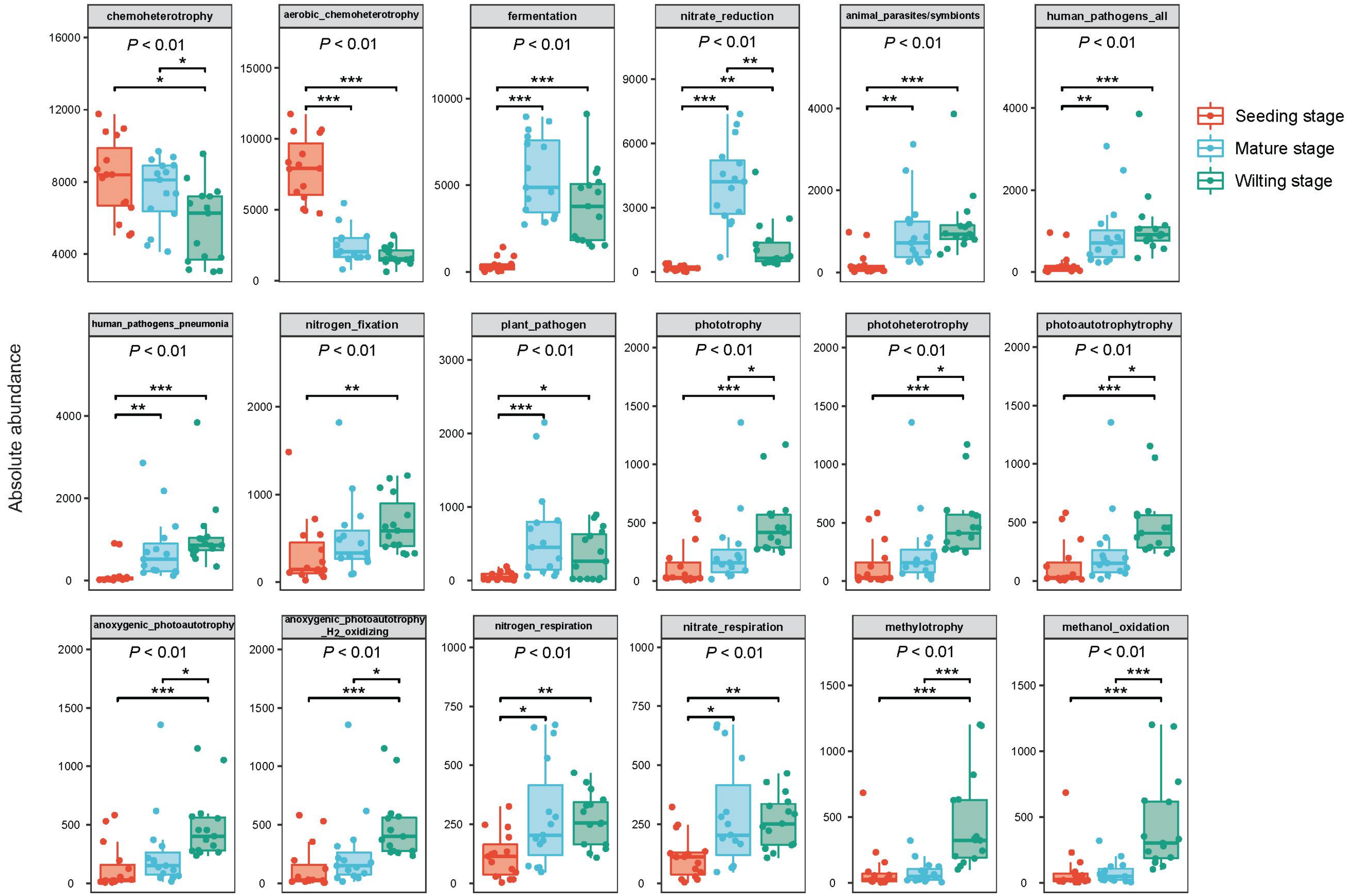

Supplementary Fig. 5 The The variation analysis of functional annotation of bacterial communities in root endosphere based on FAPROTAX across developmental stages

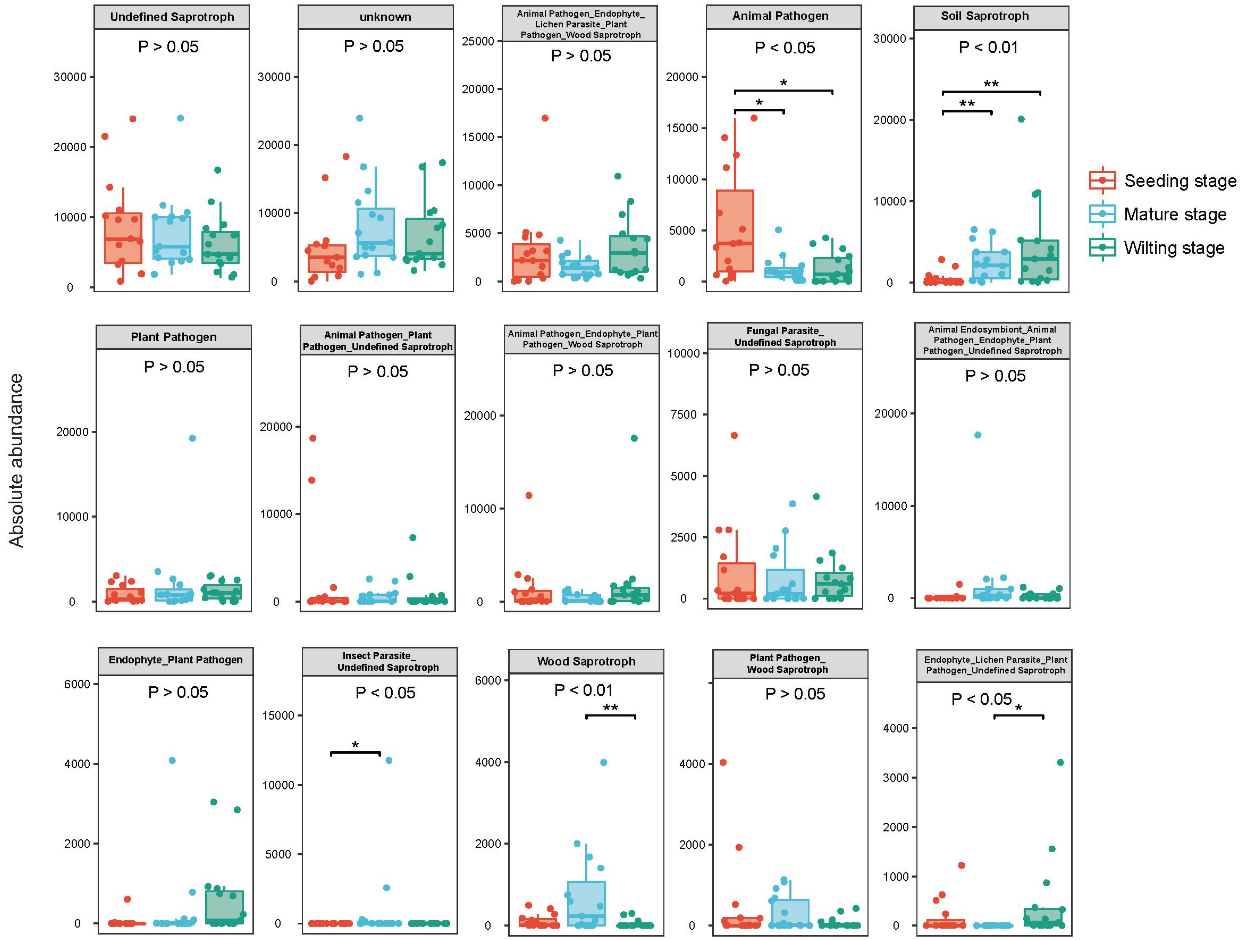

Supplementary Fig. 6 The The variation analysis of functional annotation of fungal communities in root endosphere based on FUNGuild across developmental stages
